# O-35 - USING ARTS BASED APPROACHES TO INCREASE ENGAGEMENT IN TRAVEL HEALTH VACCINE PREVENTABLE DISEASE

**DOI:** 10.1093/pubmed/fdag046.031

**Published:** 2026-07-09

**Authors:** Miss Jane Beresford, Ms Jackie Sands

**Affiliations:** NHS Greater Glasgow & Clyde; NHS Greater Glasgow & Clyde

## Abstract

**Background:**

The removal of travel health risk assessments and vaccinations from General Practice has made it more difficult to reach leisure and VFR travellers, particularly within South Asian and African communities in NHS Greater Glasgow and Clyde. This has led to reduced awareness of, and access to, the typhoid vaccine, increasing infection risk and the potential for health inequalities.

Arts-based engagement offers a culturally appropriate and accessible way to share health information. By working with trusted artists and community peers, it supports two-way communication, counters misinformation, builds trust, and positively influences vaccine attitudes and motivation. These approaches can also help reduce barriers related to access and confidence, enabling more informed vaccination decisions.

**Objectives:**

To develop a co-production approach using the Arts

To develop a communications strategy to promote the service to marginalised populations

Improved understanding and uptake of Travel Health Service

**Methods:**

NHS GGC partnered with GAMSCA (Gambians in Scotland Community Association), a Glasgow-based organisation supporting the Gambian and wider African community, to deliver arts-based workshops using Batik printing. The workshops explored awareness of travel health, the need for protection when travelling, typical actions taken by individuals and families, risks of vaccine-preventable diseases, and awareness of current travel health service provision.

**Results:**

The findings showed low awareness of travel health. Participants believed they did not need protection when travelling to their country of birth.and reported becoming unwell during visits. Discussions highlighted the importance of travel risk assessments and destination-specific vaccinations in preventing infections.

**Conclusions:**

The workshops generated interest and trust, with discussions on vaccines, confidence and conspiracy theories. The team addressed concerns. Participants were engaged through arts-based *Travel Safe* activities, which revealed low awareness of the Travel Health Service and protection when travelling to countries of birth. The use of arts built trust and engagement. An evaluation is underway.

